# Caloric restriction: is mammalian life extension linked to p53?

**DOI:** 10.18632/aging.100481

**Published:** 2012-08-22

**Authors:** Paola Tucci

**Affiliations:** ^1^ Medical Research Council, Toxicology Unit, Leicester University, Leicester LE1 9HN, UK; ^2^ Department of Pharmaco-Biology, University of Calabria, 87036 Rende (CS), Italy

**Keywords:** p53, senescence, aging, caloric restriction, apoptosis, cell death, cell cycle

## Abstract

Caloric restriction, that is limiting food intake, is recognized in mammals as the best characterized and most reproducible strategy for extending lifespan, retarding physiological aging and delaying the onset of age-associated diseases. The aim of this mini review is to argue that p53 is the connection in the abilities of both the Sirt-1 pathway and the TOR pathway to impact on longevity of cells and organisms. This novel, lifespan regulating function of p53 may be evolutionarily more ancient than its relatively recent role in apoptosis and tumour suppression, and is likely to provide many new insights into lifespan modulation.

## INTRODUCTION

Caloric restriction (CR), that is limiting food intake, is recognized in mammals as the best characterized and most reproducible strategy for extending lifespan, retarding physiological aging and delaying the onset of age-associated diseases [1]. Restricted calorie intake modifies the rate of aging and cellular pathology, reduces the age-associated accumulation of oxidatively damaged proteins, lipids and DNA and also prevents many of the changes in gene expression and transcriptional activity that normally occur with ageing [1]. Although several theories have been advanced over the years to explain the anti-ageing effects of CR, one favoured hypothesis is that CR acts by decreasing oxidative stress [2]. Biologically, different animal species are characterised by markedly different lifespan. For example, mice have relatively short (around 2-year) mean longevity, whereas humans live to a mean of 70-80 years. Animals with higher metabolic rates often have shorter life spans. The higher the metabolic rate of an organism, the greater the production of reactive oxygen species (ROS) and hence the shorter the life span; however, in some species the strict correlation between metabolic rate and life span is not maintained. Birds and primates, for example, tend to live longer than would be predicted by their metabolic rates. This is because, at a given metabolic rate, mitochondria from these species tend to produce fewer ROS. Thus the accumulation of molecular damage and the resulting increase of oxidative stress caused by ROS was thought to contribute to aging rather than metabolic rate itself and provided the strongest correlation with overall longevity [3]. More recently, however, became evident that the ROS model cannot be the only driving-force of aging. No animal lives long enough to experience the consequences of damage by ROS, since other factors terminate its life first [4]. Further, the overexpression of major antioxidant enzymes, which decrease free radicals, does not extend the lifespan of mice [5], and superoxide dismutases, the major reactive oxygen species regulating autophagy [6], protect against oxidative stress but have little or no effect on life span in C. Elegans [7].

Thus despite impressive progress in identifying the key components of the CR pathway, many proximal effectors of CR induced longevity remain unknown to date. This can be related to the fact that CR causes a myriad of transcriptional and physiological changes that are related to its positive effects on lifespan [8-12]. Are these benefits due to passive effects of lowered caloric intake or is it the result of a highly evolved and regulated process?

The aim of this short review is to argue that CR is indeed a regulated process and that its main regulator pathways, mTOR and Sirt-1, are connected to the p53 pathway.

### The mTOR/Sirt-1 pathway

The CR-driven reduction of metabolic rate involves downregulation of the nutrient sensor mammalian target of rapamycin (mTOR), the evolutionarily conserved serine/threonine protein kinase that is strongly involved in most cellular functions and implicated in stimulating cell growth [13-21]. The IGF-1–AKT–TOR network is an evolutionarily conserved pathway that transmits survival signals in cells in response to growth factor stimulation. mTOR is able to regulate both apoptosis and autophagy, and therefore affects the fate of cells. Indeed, apoptosis is clearly implicated in cancer [22-55] as well as in neurodegeneration [56-63]. The binding of a growth factor (IGF-1) to its tyrosine kinase receptor (IGF-1R) results in the recruitment and activation of PI3 kinase to the plasma membrane receptor, which in turn phosphorylates the inositides, increasing the local concentration of PIP3 and PIP2 at the plasma membrane. This increase in lipid second messengers recruits and activates the PDK and AKT protein kinases at the plasma membrane where AKT is then fully activated by phosphorylation of ser-473 and thr-308 [64-71]. AKT has several substrates that are antiapoptotic such as FOXO, BAD [72-73] and MDM2 [74]. In addition the activated AKT protein moves to the cell nucleus where it phosphorylates the forkhead transcription factors. These events result in a program leading to antiapoptotic signalling, preparation for entry into the cell cycle and cell growth, and communication with the TOR kinase pathway, which senses nutrient levels (glucose and amino acids) in the environment. This is accomplished by AKT-1 phosphorylation and inactivation of TSC2 [75-78], which forms a TSC1–TSC2 protein complex that is a GAP for the RHEB G-protein. RHEB, in turn, activates the TOR kinase [79-81]. Thus, an active AKT-1 activates the TOR kinase, both of which are positive signals for cell growth (an increase in cell mass) and division. Furthermore, CR, which reduces the levels of insulin and IGF-1 in serum, has been shown to extend life span and delay the onset of age-associated pathologies through inhibition of TOR [82-85].

The absence of glucose in the cell also increases the levels of AMP, a coactivator of AMPK. Active AMPK positively regulates the activity of the TSC1–TSC2 complex by phosphorylating the TSC2 protein (resulting in the opposite activity to the AKT-1 phosphorylation of TSC2), which then turns off the RHEB G-protein and reduces TOR activity [86].

**Figure 1:**
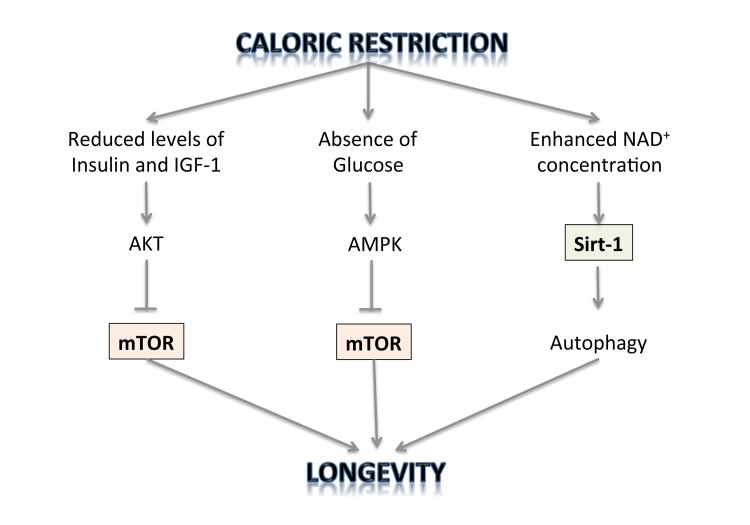
The regulation of longevity by caloric restriction.

The TOR kinase regulates two processes that can account for the observed effect of dietary restriction on longevity: translation of selected mRNAs in the cell and autophagy. The first is the rate of protein synthesis, which is modulated by the effect of TOR on the ribosomal protein S6 kinase (S6K) and on the translation initiation factor 4E-binding protein (4E-BP) [87]. It is therefore possible that inhibition of TOR just leads to reduction in the rate of protein synthesis and this is the mechanism of its lifespan prolonging activity. Inhibition of translation may shift cell metabolism to a physiological state that favours maintenance and repair and this may lead to extension of lifespan. Regulation of autophagy is another process by which TOR may affect cell longevity [88]. Accumulating evidence demonstrates that longevity pathways interact with the autophagic process to regulate diverse cellular functions including growth, differentiation, response to nutrient deprivation and oxidative stress, cell death, as well as macromolecule and organelle turnover. This entails the formation of double-membrane vesicles in the cell cytoplasm that engulf cytoplasmic components, including defective mitochondria, and move them to the lysosomal compartment where they are degraded. Autophagy can be induced by stress and also by caloric restriction. The mechanisms by which enhanced autophagy can improve organismal health and longevity are largely elusive. As one possibility, increased autophagy might improve cellular resistance to stress by augmenting the metabolic buffering capacity of cells. Alternatively, autophagy might enhance organellar turnover and mediate a “cleaning effect”, thereby preventing the accumulation of damaged/old organelles [89-105]. But only physiological levels of autophagy can promote survival under stressful conditions. Reduced autophagy may be an oncogenic event and contribute to tumour progression, while enhanced autophagy is activated in tumour cells in which the availability of oxygen and nutrients is poor and represents an adaptive survival mechanism to overcome drug-induced cellular stress and cytotoxicity [106-116]. Thus autophagy is negatively regulated by mTOR, whose activity can be inhibited by rapamycin and caloric restriction [117-124]. Thus, it has been shown that CR slows down aging through inhibition of TOR. On the other hand, it has also been demonstrated that CR activates NAD(+)-dependent deacetylases, called sirtuins, known to be involved in aging and age-related diseases, thus extending lifespan [125-127] (Figure 1). The Sirt-2 gene was shown to regulate life span in yeast; increased dosage extended life span, and loss of function shortened it [128]. A similar relationship exists between the Sirt-2 gene in C. elegans and life span [129]. Because of the enormous evolutionary divergence between yeast and C. elegans, it is likely that Sirt-2-related genes determine the life span in a broad spectrum of organisms, including mammals. Caloric restriction/nutrient deprivation stimulates autophagy through the activation of the mammalian Sirt-1 gene [130-135]. Indeed, Sirt-1 functions as a metabolic sensor that detects the increase in NAD+ concentrations resulting from enhanced NADH oxidation. Once activated, Sirt-1, which is an NAD-dependent HDAC, can deacetylate essential autophagic modulators and may affect cellular pathways involved in glucose homeostasis.

But which pathway is indispensable for prolonged lifespan by CR, inhibiting TOR or activating sirtuins? Evidence has emerged that sirtuins and mTOR are involved in the same longevity pathway [136]. Importantly, resveratrol, an activator of sirtuins, antagonizes the mTOR/S6K pathway [137]. Therefore, the two notions that CR prolongs lifespan either by activating sirtuins or by deactivating TOR are, in fact, complementary: CR deactivates the mTOR pathway in part by activating Sirt-1.

### The connection with p53

The p53 protein and its encoding gene were first identified in 1979 because of its association with cancer [138-141] and its function as a tumour suppressor gene [142-168]. In response to various stress signals, p53 selectively regulates a set of its target genes and initiates various stress responses, including cell cycle arrest, apoptosis, and/or senescence, to exert its function in DNA damage and tumour suppression. In addition, p53 may play a dual role in autophagy regulation. On the one hand, nuclear p53 can induce autophagy promoting the transcription of proapoptotic and cell cycle-arresting genes. In contrast, cytoplasmic p53 degradation exerts an autophagy-inhibitory function [169]. Loss of p53 thus provides two levels of growth advantage to tumour cells; it removes two mechanisms of eliminating the cell in response to genotoxic stress, and at the same time, enables cell survival under limiting nutrient conditions. Paradoxically, each of these exploits the same process, autophagy, utilizing its opposing functions.

The existence of p53 in short lived organisms that do not develop adult cancers, such as flies and worms, suggests that tumour suppression is not the only or, indeed, the original function of p53. Indeed, recent studies have shown that p53 and its family members, including p63 and p73, all play important roles in reproduction [170,171]. Emerging evidence has suggested that p53 is also an important but complex player in the regulation of aging and longevity in worms, flies, mice, and humans. The impact of p53 on aging and longevity in humans has been recently indicated by several epidemiological studies [172,173]. Moreover longevity is always coupled with the age of attaining reproductive maturity in animals. The later in life that reproductive maturity occurs, the greater the longevity of that animal. Animals will most often not reproduce in times of stress and starvation of nutrients and will shut down their reproductive processes. Indeed, p53 in adult worms and flies is predominantly localized in the germline where it is employed in the prevention of reproduction in response to stress signals such as DNA damage and starvation. Thus p53 has its origins, in an evolutionary sense, as a germline surveillance molecule under conditions of starvation or DNA damage [171]. It is only in vertebrates, where the body plan requires self-renewal of tissues (flies and worms are largely post-mitotic as adults, except for the germline), where the p53 protein is found in somatic tissues and takes on the function of a tumour suppressor.

**Figure 2:**
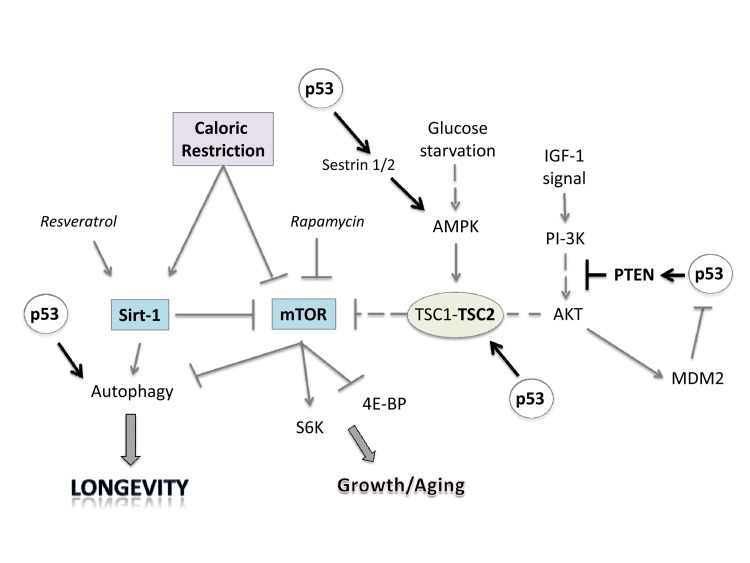
Linking caloric restriction (CR), sirtuins, and mTOR to the p53 pathway. CR and resveratrol activate autophagy through sirtuins, thus extending lifespan. The intracellular mTOR pathway via inputs of PI-3K, AMPK and other sensors integrates nutrient availability and drives cell growth and aging. Rapamycin and resveratrol inhibit the Sirtuin/mTOR network. CR and p53 may also inhibit mTOR activity through upregulation of known negative regulators PTEN, TSC2 and AMPK. The products of two p53 target genes, Sestrin 1 and 2 activate AMPK, which phosphorylates TSC2 and stimulates its GAP activity enabling mTOR inhibition. Glucose starvation inhibits mTOR by promoting TSC1/2 activation.

p53 interacts with IGF-1, TOR and Sirt-1 pathways, the critical pathways that regulate aging and longevity [174,175] (Figure 2). There are two major connections between the proteins of these three pathways that form a rapid and a slower response to stress signals after activation of p53. First, the rapid signal transduction pathway responds to DNA damage by the activation of p53 and AMPK, which in turn activates TSC2 via phosphorylation [176,177]. This inactivates RHEB and then mTOR and shuts down translation while turning on autophagy. These events are p53 dependent in a cell, as well as TSC1–TSC2 dependent after DNA damage, as demonstrated by using cells that had no p53, TSC1, or TSC2 genes (from knockout mice). A different stress signal such as glucose starvation rapidly activates this pathway involving p53 and AMPK. AMPK can induce p53 by promoting phosphorylation on serine-15, a site known to be important for the activation of p53 [176]. There is a second wave of communication between p53 and the IGF-1–AKT and TOR pathways. Thus, Mak et al. [178] first demonstrated that PTEN was induced by the activation of p53. Cell lines or mouse tissues that transcriptionally activated the PTEN gene after DNA damage also induced TSC2 gene transcription. The p53-mediated induction of PTEN and TSC2 acts in the same way as the faster p53–AMPK pathway. Increasing PTEN levels shuts down AKT activity and relieves its inhibition on TSC2, resulting in the inactivation of TOR, loss of phosphorylation of S6 kinase, and activation of autophagy [176,179]. Similarly, the activation of AKT-1 and mTOR by the presence of nutrients and growth factors leads to the AKT-1-dependent activation of MDM2 by phosphorylation, which enhances its activity as a ubiquitin ligase and moves it into the nucleus so that it more effectively degrades and inactivates p53 [180].

### Conclusion

**Figure 3:**
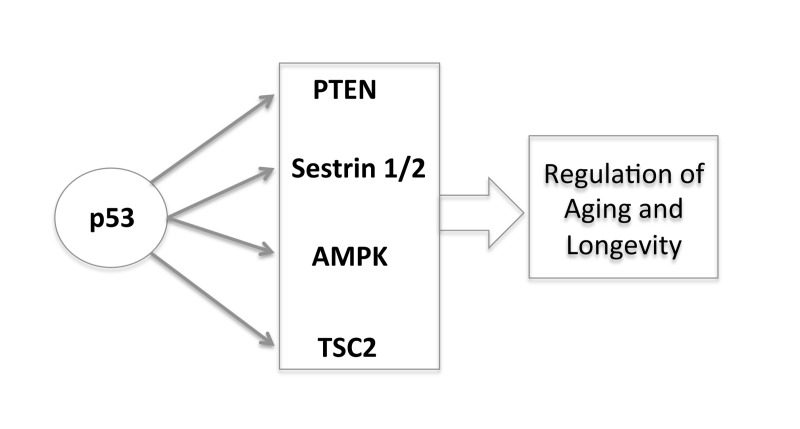
The new and complex role of p53 in regulating aging and longevity through the transcriptional regulation of different target genes.

Here, we have reviewed the role of caloric restriction in longevity and argued that p53 is the connection in the abilities of both the Sirt-1 pathway and the TOR pathway to impact on longevity of cells and organisms. Furthermore the integration of the p53 pathway with the IGF-1 and TOR pathways brings together a number of overlapping concepts that play a central role in life processes. Through the transcriptional regulation of different target genes (PTEN, AMPK, Sestrin 1/2, TSC2) p53 negatively regulates the insulin/IGF-1 and TOR signaling, creating an interpathway network that permits cells to inhibit cell growth and division to avoid the introduction of errors during these processes under stress conditions (Figure 3). In this way, p53 increases the fidelity of these processes over the lifetime of an organism. Since decreased TOR/insulin/IGF-1 signaling extends life span, p53 may regulate aging and longevity through its down-regulation of the signaling of these two critical pathways.

This novel, lifespan regulating function of p53 may be evolutionarily more ancient than its relatively recent role in apoptosis and tumour suppression, and is likely to provide many new insights into lifespan modulation.

## Abbreviations

CR: caloric restriction
ROS: reactive oxygen species
mTOR: mammalian target of rapamycin
IGF-1: insulin-like growth factor-1
IGF-1R: IGF-1 receptor
